# *Carica papaya* Leaves Juice Significantly Accelerates the Rate of Increase in Platelet Count among Patients with Dengue Fever and Dengue Haemorrhagic Fever

**DOI:** 10.1155/2013/616737

**Published:** 2013-04-11

**Authors:** Soobitha Subenthiran, Tan Chwee Choon, Kee Chee Cheong, Ravindran Thayan, Mok Boon Teck, Prem Kumar Muniandy, Adlin Afzan, Noor Rain Abdullah, Zakiah Ismail

**Affiliations:** ^1^Bioassay Unit, Herbal Medicine Research Center, Institute for Medical Research, Jalan Pahang, 50588 Kuala Lumpur, Malaysia; ^2^Department of Internal Medicine, Tengku Ampuan Rahimah Hospital, Jalan Langat, 41200 Klang, Malaysia; ^3^Epidemiology and Biostatistics Unit, Institute for Medical Research, Jalan Pahang, 50588 Kuala Lumpur, Malaysia; ^4^Virology Unit, Infectious Disease Research Center, Institute for Medical Research, Jalan Pahang, 50588 Kuala Lumpur, Malaysia

## Abstract

The study was conducted to investigate the platelet increasing property of *Carica papaya* leaves juice (CPLJ) in patients with dengue fever (DF). An open labeled randomized controlled trial was carried out on 228 patients with DF and dengue haemorrhagic fever (DHF). Approximately half the patients received the juice, for 3 consecutive days while the others remained as controls and received the standard management. Their full blood count was monitored 8 hours for 48 hours. Gene expression studies were conducted on the ALOX 12 and PTAFR genes. The mean increase in platelet counts were compared in both groups using repeated measure ANCOVA. There was a significant increase in mean platelet count observed in the intervention group (*P* < 0.001) but not in the control group 40 hours since the first dose of CPLJ. Comparison of mean platelet count between intervention and control group showed that mean platelet count in intervention group was significantly higher than control group after 40 and 48 hours of admission (*P* < 0.01). The ALOX 12 (FC  =  15.00) and PTAFR (FC  =  13.42) genes were highly expressed among those on the juice. It was concluded that CPLJ does significantly increase the platelet count in patients with DF and DHF.

## 1. Introduction

Malaysia is blessed with 12000 species of flowering plants of which 1300 have medicinal properties [1]. There is a rapidly growing response to the use of medicinal plants by the Malaysian population. WHO estimates that in many countries 80% of the rural patients seek alternative treatment using medicinal plants. 

*Carica papaya* is a member of the Caricaceae and is a dicotyledonous, polygamous, and diploid species [2]. It originated from Southern Mexico, Central America, and the northern part of South America. It is now cultivated in many tropical countries such as Bangladesh, India, Indonesia, Sri Lanka, the Philippines, and the West Indies including Malaysia. Malaysia is known to be one of the top 5 papaya exporting countries [3]. The papaya fruit is globally consumed either in its fresh form or the form of juices, jams, and crystallized dry fruit [4]. The ripe fruit is said to be a rich source of vitamin A, C, and calcium [5]. There are many commercial products derived from the different parts of the *C. papaya* plant, the most prominent being papain and chymopapain, which is produced from the latex of the young fruit, stem, and the leaves. *C. papaya* leaves have been used in folk medicine for centuries. Recent studies have shown its beneficial effect as an anti-inflammatory agent [6], for its wound healing properties [7], antitumour as well as immune-modulatory effects [8] and as an antioxidant [9]. A toxicity study (acute, subacute, and chronic toxicity) conducted on *Sprague Dawley* rats administered with *Carica papaya* leaves juice (CPLJ) of the sekaki variant revealed that it was safe for oral consumption [10].

Dengue is an arthropod-borne viral disease carried by *Aedes aegypti* as the vector, caused by 4 possible viral serotypes, namely, serotype 1, 2, 3, and 4 of the Flaviviridae family. In Malaysia, dengue cases have been on the rise since 2002. Total of 18,371 cases of dengue fever (DF) and dengue haemorrhagic fever (DHF) were reported last year and had claimed 33 lives in the same year [11]. There is no specific antiviral drug available for the treatment of dengue infection. Infected patients receive supportive management with fluids, blood and blood, products complying to the Ministry of Health Clinical Practice Guidelines (CPGs) on Management of Dengue, 2010. Each episode of infection is known to induce a life-long protective immunity to the homologous serotype but confers only partial and transient protection against subsequent infection by the other serotypes. Secondary infection is a major risk factor for DHF possibly due to antibody-dependent enhancement. A patient with dengue fever presents typically with fever, headache, and rash known as the dengue triad. There are many other nonspecific signs and symptoms associated with DF and patient can progress to DHF and typically manifests as abdominal pain, bleeding, and even circulatory collapse. The clinical course of dengue has an abrupt onset followed by three phases, namely, the febrile phase, the critical phase and the recovery phase. It is during the critical phase that thrombocytopaenia, characterized by a decrease in platelet count below 100 000 per mm^3^ from the baseline and haemoconcentration, characterized by an increase of haematocrit by 20% or more, is detectable before the subsidence of fever and the onset of shock [12]. 

Certain genes have been shown to influence platelet production and platelet aggregation, namely, the Arachidonate 12-lipoxygenase (ALOX 12) also known as the Platelet-type Lipoxygenase as well as the Platelet-Activating Factor Receptor (PTAFR). An increase in activity of these genes is required for platelet production and activation. The ALOX 12 gene is strongly expressed in megakaryocytes and has been known to be responsible for the 12-Hydroxyeicosatetraenoic acid (12-HETE) production of platelets [13]. The PTAFR gene was been found to be expressed in megakaryocytes indicating that it could be a precursor for platelet production in addition to its well known role in platelet aggregation [14]. 

Safety studies based on OECD guidelines for acute, subacute, and chronic toxicity were conducted on *C. papaya* extract and showed that it was found to be safe for human consumption [10]. The present study was conducted to determine and investigate the traditional claim that CPLJ increases the platelet count in patients with DF and DHF.

## 2. Materials and Method

### 2.1. Plant Material and Sample Preparation

*C. papaya* leaves of the the sekaki variant were chosen for the study based on fingerprinting and safety analysis which also contained allowable limit of heavy metals and microbial content. For the purpose of the study, a private plantation certified by the Ministry of Agriculture in Semenyih, Selangor was identified to provide the leaves for the entire duration of the study to ensure similar source of authenticated raw material used. The trees in this plantation were kept free of herbicides, pesticides, and insecticides. Juice was prepared fresh from the leaves that were washed thoroughly with an organic vegetable cleaning agent and reverse osmosis water few times. Juice was extracted from 50 grams of fresh leaves using a juice extractor without any addition of water, under sterile conditions. The fresh juice was aliquoted at a volume of approximately 30 mLs in sterile glass vials and transported daily in an icebox and kept at a temperature of below 4°C to the study site at the male and female dengue wards of Hospital Tengku Ampuan Rahimah, Klang, Selangor.

The juice was characterized and standardized using a High Performance Liquid Chromatography Diode Array Detector according to three markers: manghaslin, clitorin, and rutin. The chemical fingerprinting of the leaves was consistent throughout the study.

### 2.2. Subjects

An open labeled randomized controlled trial was conducted to provide clinical data in support of the proposed claims. The number of patients involved was determined based on the following calculation.

With plan to have a continuous response variable from independent control and experimental subjects with 1 control(s) per experimental subject, expecting the platelet count to increase by 20,000 after administration of CPLJ for three consecutive days, setting the standard deviation for platelet count at 40,000 in a normal population [15], type I error (alpha) = 0.01 and power of study of 90%, the sample size calculated by using PS software [16] was 242 with each arm of 121 intervention group and control group. Anticipating dropout rate to be 20%, the final sample size was 290 (145 each for intervention and control group).

All the patients meeting the inclusion criteria were recruited from the Dengue Ward of Hospital Tengku Ampuan Rahimah, Klang, Selangor Malaysia. Subjects were randomly assigned to either intervention or control group using the block of 10 method. This method was used to ensure that an equal number of participants were allocated to interventional and control groups. Therefore, with the block size of ten, of every ten consecutively enrolled participants, five will be allocated to one interventional group and five to the control group. For the randomization, 10 identical opaque envelopes with five envelopes each contained a card labelled as “interventional group” and other 5 envelopes each contained a card labelled as “control group.” These envelopes were given to the field investigator during the patient enrolment. The field investigator then blindly selected an envelope for each patient thus assigning the patient to interventional or control groups. This procedure was repeated until the targeted sample size was achieved.

The inclusion and exclusion criteria were used to select patients who volunteered to be enrolled into the study. Inclusion criteria were (a) male and female patients above 18 years and below 60 years old, (b) all patients who could understand or read Bahasa Malaysia or English irrespective of race, (c) patients who were confirmed to have DF or DHF grade I and II, (d) patients with a platelet count of less than or equal to 100,000/*μ*L, (e) patients with a baseline alanine transaminase (ALT) level of not more than 3 times of the upper limit of the normal range (not more than 165 U/L), and (f) patients with a creatinine kinase (CK) value of less than 500 U/L.

The exclusion criteria were (a) dengue hemorrhagic fever grade III and IV, (b) pregnant or lactating women, (c) patients who have received blood or blood products transfusion during the current hospital stay, (d) patients with underlying comorbids, (e) patients who developed Hepatitis with a serum ALT level 3 times higher than the upper limit of the normal range (>165 U/L), and (f) patients with a creatinine kinase (CK) value of more than 500 U/L.

The study was conducted in accordance with the ethical principles as outlined in the declaration of Helsinki, October 2008, following approval from the Medical Review and Ethical Committee (MREC), Ministry of Health, Malaysia and was consistent with Good Clinical Practice (GCP) and applicable regulatory requirements. The National Medical Research Registry number is NMRR-09-883-4768. Written informed consent was obtained from every volunteer prior to clinical trial participation. 

### 2.3. Diagnosis of DF or DHF and Dengue Serotyping

A clinical diagnosis of DF and DHF was made by the clinician based on patients' presentation and blood investigations. In addition a rapid dengue bedside test (SD Dengue Duo NS1 Ag + Ab Combo, Standard Diagnostics, Korea) was used to determine dengue status before the subjects were given the *Carica papaya* leave juice. This test is able to detect the presence of NS1 antigen or dengue IgM or IgG antibodies. However, for ease of interpretation of dengue status only patients who had either NS1 or IgM or both detected were included in the study. The detection of the NS1 antigen or IgM antibodies within the first five to 7 days of onset of symptoms usually indicates a current dengue infection. Subsequently, all the samples were subjected to multiplex real time RT-PCR for determination of dengue serotypes. 

### 2.4. Treatment of the Subjects

Once a current dengue infection was confirmed, a thorough screening of the patient was conducted. Baseline investigations included full blood count, bleeding profile, renal as well as liver function test, and cardiac enzymes. Patients in the intervention group received fresh juice from 50 grams of *C. papaya* leaves, once daily, 15 minutes after breakfast for 3 consecutive days while receiving the standard management as per the National Clinical Practice Guidelines for the Management of Dengue. The controls received the standard management.

### 2.5. Study Parameters

Full blood count was monitored 8 hours for the first 48 hours during the study, to determine the changes in platelet count and haematocrit levels while bleeding profile, renal profile, and liver function test were monitored daily to ensure the safety of the juice. The haematological and biochemical tests were conducted by the Pathology Department at Hospital Tengku Ampuan Rahimah, Klang and the validated results were later traced and recorded. Mean and standard deviation of the baseline haematological and biochemical parameters were then calculated as shown in Table 1. 

Blood for RNA extraction was taken on day 3 after the last dose of the juice to conduct expression studies on the PTAFR and the ALOX 12 genes. RNA Later by Ambion (United States of America) was added to the whole blood in an EDTA tube to prevent degradation of RNA.

The RNA extraction was carried out at the Institute for Medical research using an RNA extraction kit by Ambion. The RNA concentration and purity were determined using the Nanodrop 2000 Spectrophotometer by Thermo Scientific. The RNA was then converted to cDNA using the High Capacity RNA to cDNA kit by Applied Biosystems. The cDNA concentration was then determined using the Nanodrop 2000 Spectrophotometer by Thermo Scientific.

Gene expression was determined using gene expression assays by Applied Biosystems on the ABI 7500 Fast System on 12 RNA samples from the experimental group and 12 RNA samples from the control group. Specific predesigned MGB probes were used for ALOX 12 and the PTAFR gene. The 18S Ribosome was used as an endogenous control. A 10 *μ*L reaction volume was used using the TaqMan Fast Universal PCR Mastermix (2X). The probe product ID and sequence used were ALOX 12 (Hs 00167524_M1, NM_000697.2, 5′-FAM-ATTGCCATCCAGCTCAACAAATCC-MGB-3′) and PTAFR (Hs 00265399_S1, NM_001164723.1, 5′-FAM-GCCCGTAATTTATCGCGCTTACTAT_MGB-2′). 

### 2.6. Statistical Analysis

Repeated measure ANCOVA was used to determine the effect of CPLJ on the mean platelet count over 48 hours (time effect). Multiple comparisons of mean platelet count 8 hours after admission with mean platelet count at the 16, 24, 32, 40, and 48 hours after admission for interventional and control group were performed. Multiple paired *t*-test was conducted to demonstrate if there is any significant different in mean platelet count for each comparison. Hence, Bonferroni correction was applied to reduce the possibility of rejecting a true null hypotheses (commit a type 1 error). Therefore, Bonferroni correction was used to adjust the level of significance to *P* < 0.01 (0.05/number of comparison = 0.05/5 = 0.01). The efficacy of treatment of CPLJ (treatment effect) and treatment over time (time-treatment interaction) in increasing the mean platelet count was analysed by comparing mean difference in platelet count between interventional and control group. All the statistical analyses were done using PASW 18.0 (SPSS Inc., Chicago, USA). The comparative C_T_ method was used to determine the relative quantification of the genes expressed. 

## 3. Results

A total of 145 patients were recruited into the interventional group while 145 patients were recruited into the control group. At the end of the study, 111 patients from the interventional group and 117 controls were included in the statistical analysis. Sixty-two patients were excluded from the analysis as 38 patients were lost to followup and 24 patients had incomplete data (missing results due to sample rejection).

Table 1 shows demographic characteristics and baseline biochemistry investigation of respondents by treatment. In terms of dengue status, all patients recruited had either dengue NS1 or IgM or both detected, while the percentage distribution of the dengue serotypes among them was DEN1 (30.4%), DEN2 (28.4%), DEN 3 (20.6%), and DEN 4 (20.6%). Hence, all serotypes were well represented in the study.

Table 2 presents the multiple comparisons of mean platelet count 8 hours after admission with mean platelet count at 16, 24, 32, 40, and 48 hours after admission for interventional and control group Multiple paired *t*-test was conducted to demonstrate if there was any significant difference in mean platelet count for each comparison. Hence, Bonferroni correction was applied to reduce the possibility of rejecting a true null hypothesis (committing a type 1 error). Based on the number of patients recruited with complete data (111 patients from the intervention group and 117 control), the power of study was 87.0% (standard deviation of platelet count of 40,000, type I error probability of 0.01, and the true difference in mean platelet count of 20,000 between the intervention and control group). Overall, there was a significant increase in mean platelet count over 40 hours in both groups (Wilk's Lambda = 0.939, *P* = 0.015, effect size = 0.06, and power = 84.0%) after adjusting for age. Further analysis by using multiple paired *t*-test on each of the groups showed that there was a significant increase in mean platelet count at 40 hours compared to 8 hours after intervention in the intervention group (*t* = −4.256, *P* ≤ 0.001) but not in the control group (*t* = −2.399, *P* = 0.018) after adjustment of Bonferroni correction (*P* = 0.05/5 = 0.01).

Table 3 presents the overall efficacy of CPLJ supplementation by comparing the mean platelet count of interventional and control group regardless the time period. Study on the treatment effect of CPLJ on platelet count regardless of time did not show any significant difference in mean platelet count between intervention and control group (*F* = 1.128, *P* = 0.289). However, analysis of the effect of CPLJ over the study period (time and treatment effect) showed that there was a significant interaction between treatment groups and time (Wilk's Lambda = 0.934, effect size = 0.06, and power = 87.0%). 

**Figure 1:**
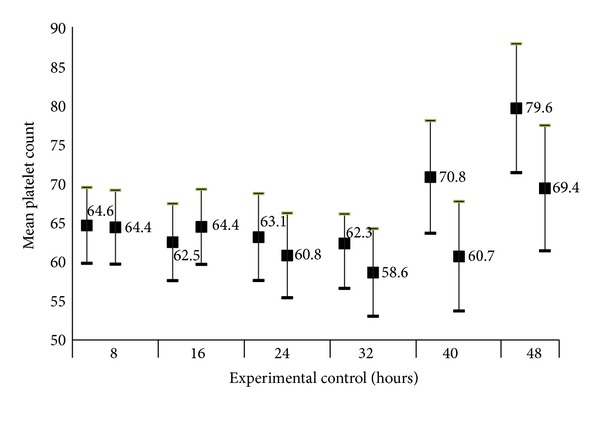
Comparison of mean platelet count between intervention and control group based on time (time-treatment effect).

Figure 1 shows the time-treatment effect of CPLJ. The intervention group had a significantly higher mean platelet count than control group at 40 hours and 48 hours of intervention.

Data from the gene expression study were analyzed using the “PCR Array Data Analysis versus 3.3” software, recommended by Applied Biosystems and showed that ALOX12 (ΔCT mean = 16.02, FC = 15.00) and PTAFR genes (ΔCT mean = 14.87, FC = 13.42) were found to be highly expressed in the intervention group when compared to the control group.

## 4. Discussion

The study conducted shows that there is a rationale behind the use of CPLJ in the treatment of some of DF and DHF. It is definitely worth investigating this plant for its potential medicinal benefits. With rapid urbanization and global travel leading to drastic demographic changes, dengue is a threat to almost 40% of the world's population. There is still no specific treatment for dengue. Previous attempts to identify a potential antiviral for the treatment of dengue has been faced with several challenges such as the presence of four distinct viral serotypes which frequently undergo mutations, finding an appropriate model for infection and protective action of a given drug as well as yield interesting therapeutic avenues for tailored response modifier drugs. The current available mouse model (AG129) available has its limitations such as low viral load and a short period of viraemia. The journey to drug discovery through the study of immune-modulatory effects against dengue infection lies on the research of generic compounds and natural products [17]. 

Research groups around Asia have attempted to study the efficacy of CPLJ in rapidly increasing platelet counts in DF as well as DHF induced thrombocytopenia but there has been no conclusive evidence drawn from those studies. Dengue is generally a self-limiting disease and the disease induced thrombocytopenia usually reverses itself after taking a slight dip during the phase of defervescence. However, a significant number of patients succumb to the disease during the thrombocytopenic period. Many mechanisms come into play during the critical phase of the disease to help reverse the disease state at this point. Animal studies in elucidating safety data have been conducted on normal *Sprague Dawley* rats using freeze dried CPLJ; however, no significant increase in platelet count was observed among the rats given the juice and the rats kept as control [10]. This was probably due to the fact that the juice was freeze dried and certain essential compounds could have been lost during the process of freeze drying or perhaps the right disease model was not used for the study. Haematocrit level, which is an important parameter which is usually monitored to determine the rate of improvement in haemoconcentration, was found to be significantly reduced in both groups of people. White Blood cell count which is found to be reduced in viral infections was also found to increase in both groups.

The RNA was extracted from the blood of the patients recruited and gene expression of two genes, namely, the ALOX 12 and the PTAFR which were conducted so far. There was a 15-fold increase in the ALOX 12 gene activity among the patients in the experimental group as compared to those in the control group at the end of the 3 days. ALOX 12 is known to be associated with increased megakaryocyte production as well as its conversion to platelets through 12-HETE mediated pathway which in turn leads to increased platelet production. A study was conducted at the Royal College of Surgeons, Ireland, to determine the platelet specific genes. The Alox 12 gene was highly expressed in platelets and found to be a platelet specific gene by McRedmond et al. [18]. A study conducted in Temple University School of Medicine, Philadelphia provided evidence that ALOX12 is a direct target of transcription factor RUNX1 in megakaryocytes and platelets. RUNX1 is a transcription factor that regulates the expression of haemopoietic-specific genes. When there is RUNX1 haplodeficiency, it affects overall haemopoiesis and hence, ALOX 12 expression in platelets is decreased. There was also an agonist-induced decreased 12-HETE production in platelets with the decrease in ALOX 12 expression. This provides further evidence that platelet production is associated with ALOX 12 expression [13]. 

This finding supports the claim that the juice consumption during the course of dengue infection has the potential to induce the rapid production of platelets. This was clearly demonstrated by the significant increase in the mean platelet count after 40 hours and 48 hours of juice consumption. The PTAFR gene which is known to be responsible for increased platelet production and aggregation was expressed 13.42-folds among the patients who consumed the juice as compared to the control group indicating that the juice had played an important role in addressing the arresting of bleeding tendencies among these patients. A study conducted in Brazil showed that injection of Platelet Activating Factor (PAF/PTAFR) in mice induced an increase in platelet count. However, after a certain level, further administration of PAF failed to induce platelet production indicating autosensitization. These findings show that PAF/PTAFR can induce the release of platelets which may be relevant to thrombocytosis [19]. We are currently investigating many other genes to determine other roles of the CPLJ other than its role in platelet production and activation.

As all plants *C. papaya* leaves are rich in compounds of different properties. Further studies need to be conducted before determining the inflammatory pathways affected by the juice, unopposed. However, it can be concluded that the administration of CPLJ in DF and DHF is safe and does induce the rapid increase in platelet count. It may play a valuable role in the management of DF in the near future.

## Figures and Tables

**Table 1:** Demographic characteristics and baseline biochemistry investigation of respondents by treatment.

Demographic characteristics	Interventional group *n* (%)	Control group *n* (%)	*χ* ^2^ (df)*	*P* value
Gender				
Men	91 (46.9)	103 (53.1)	1.644 (1)	0.200
Women	20 (58.8)	14 (41.2)		
Ethnicity				
Malay	77 (48.1)	83 (51.9)		
Chinese	4 (57.1)	3 (42.9)		
Indian	11 (50.0)	11 (50.0)		
Others	19 (48.7)	20 (51.3)		
Age (mean/SD)	30.4 (10.3)^†^	26.4 (7.3)^†^	−3.370^††^	0.001
Type of dengue fever				
Classical dengue fever	42 (53.2)	37 (46.8)	0.971 (1)	0.324
Dengue haemorrhagic fever	69 (46.3)	80 (53.7)		
Baseline Haematology investigation				
Platelet count (×10^3^/*μ*L)	66.4 (22.8)^†^	69.0 (22.6)^†^	0.847^††^	0.398
Total white blood cell (×10^3^/*μ*L)	3.4 (1.7)^†^	3.3 (1.6)	−0.297^††^	0.766
Haemoglobin (g/%)	13.9 (1.5)^†^	14.2 (1.4)^†^	1.595^††^	0.112
Haematocrit (%)	41.5 (4.2)^†^	42.9 (6.0)^†^	1.878^††^	0.062
Lymphocytes (%)	41.8 (14.2)^†^	40.9 (14.8)^†^	−0.459^††^	0.646
Neutrophils (%)	42.1 (17.7)^†^	42.1 (19.4)^†^	0.017^††^	0.987

*Pearson Chi-Square. Df (degree of freedom), ^†^mean and standard deviation, ^††^ *t*-value for independent samples *T*-test.

**Table 2:** Comparison of platelet count in each group based on time.

Type of laboratory investigation	Interventional group (*n* = 111)			Control group (*n* = 117)		
Platelet count	Mean difference (95% CI)	*t*	*P* value	Mean difference (95% CI)	*t*	*P* value
8–16 hours	0.993 (−1.660, 3.645)	0.740	0.460	−1.411 (−3.961, 1.140)	−1.094	0.276
8–24 hours	−0.432 (−4.422, 3.558)	−0.214	0.831	2.213 (−0.523, 4.948)	1.600	0.112
8–32 hours	−2.716 (−7.540, 2.107)	−1.114	0.267	2.775 (−0.796, 6.347)	1.537	0.127
8–40 hours	−7.890 (−14.472, −1.310)	−2.374	0.019	0.867 (−3.472, 5.207)	0.395	0.693
8–48 hours	−16.764 (−24.566, −8.964)	−4.256	<0.001	−7.703 (−14.055, 1.351)	−2.399	0.018

Repeated measure ANCOVA within group analysis was applied followed by multiple paired *t*-tests.

*Bonferroni correction was applied by correcting the level of significance (0.05/5 = 0.01). Potential covariate (age) was controlled by using repeated measures ANCOVA (ANCOVA controlled for age).

**Table 3:** Comparison of platelets count between experiment and control group (treatment effect regardless of time).

Type of lab. Investigation	Mean difference (95% CI)	*P* value
Platelet count	2.349 (−5.151, 9.850)	0.538

Repeated measure ANCOVA between group analyses was applied. Level of significance was set at 0.05 (two-tailed).
